# Comparing atmospheric and hypoxic cultured mesenchymal stem cell transcriptome: implication for stem cell therapies targeting intervertebral discs

**DOI:** 10.1186/s12967-018-1601-9

**Published:** 2018-08-10

**Authors:** C. Elabd, T. E. Ichim, K. Miller, A. Anneling, V. Grinstein, V. Vargas, F. J. Silva

**Affiliations:** 1 Immune Advisors, LLC, La Jolla, CA 92037 USA; 2 BioRestorative Therapies, Inc., 40 Marcus Drive, Suite 1, Melville, NY 11747 USA

**Keywords:** Hypoxia, Mesenchymal stem cells, RNA sequencing, Transcriptome, Cell therapy, Orthopedic application, Intervertebral disc

## Abstract

**Background:**

Mesenchymal stem cells (MSCs) represent an attractive avenue for cellular therapies targeting degenerative diseases. MSC in vitro expansion is required in order to obtain therapeutic numbers during the manufacturing process. It is known that culture conditions impact cellular properties and behavior after in vivo transplantation. In this study, we aimed at evaluating the benefit of hypoxic culturing of human bone marrow derived mesenchymal stem cells on cell fitness and whole genome expression and discussed its implication on cellular therapies targeting orthopedic diseases such as chronic lower back pain.

**Methods:**

Human bone marrow mesenchymal stem cells (hBMMSCs) were isolated from fresh human anticoagulated whole bone marrow and were cultured side by side in atmospheric (20% O_2_) and hypoxic (5% O_2_) oxygen partial pressure for up to 3 passages. Stem cell fitness was assessed by clonogenic assay, cell surface marker expression and differentiation potential. Whole genome expression was performed by mRNA sequencing. Data from clonogenic assays, cell surface marker by flow cytometry and gene expression by quantitative PCR were analyzed by two-tailed paired Student’s t-test. Data from mRNA sequencing were aligned to hg19 using Tophat-2.0.13 and analyzed using Cufflinks-2.1.1.

**Results:**

Hypoxic culturing of hBMMSCs had positive effects on cell fitness, as evidenced by an increased clonogenicity and improved differentiation potential towards adipocyte and chondrocyte lineages. No difference in osteoblast differentiation or in cell surface markers were observed. Only a small subset of genes (34) were identified by mRNA sequencing to be significantly dysregulated by hypoxia. When clustered by biological function, these genes were associated with chondrogenesis and cartilage metabolism, inflammation and immunomodulation, cellular survival, migration and proliferation, vasculogenesis and angiogenesis.

**Conclusions:**

Hypoxic culturing positively impacted hBMMSCs fitness and transcriptome, potentially improving inherent properties of these cells that are critical for the development of successful cellular therapies. Hypoxic culturing should be considered for the in vitro expansion of hBMMSCs during manufacturing of cellular therapies targeting orthopedic disorders such as lower back pain.

## Background

The field of cell-based therapeutics is growing at a fast pace and has reached unprecedented interest with the recent FDA approval of certain chimeric antigen receptor (CAR) T-cell therapies. The use of mesenchymal stem cells (MSCs) targeted towards degenerative diseases is under extensive investigation with a number of active clinical trials around the world. MSCs are adherent to plastic with a fibroblastic morphology and have the ability to self-renew and differentiate into mesodermal lineages. Because of their ability to migrate to injury sites, immune-modulating property and their ability to differentiate in situ to repair damaged tissues, MSCs represent an attractive option for cellular therapies targeting a variety of indications [1]. MSCs can be readily isolated from many tissues, such as bone marrow, adipose tissue, skeletal muscle, dental pulp and umbilical cord [2], but require in vitro expansion in order to reach clinically therapeutic numbers [3, 4].

Determining adequate cell culture conditions is critical in order to develop effective cellular therapies. Significant effort has been put on controlling MSC behavior and fate in vitro to modulate the microenvironment of targeted tissues in order to adapt or select optimum cell populations. Donor age, plating density, passage number, extracellular matrix protein coating, mechano-/electro-stimuli and three-dimensional scaffolds have been shown to influence cell characteristics [5]. Controlling oxygen partial pressure in vitro by reducing atmospheric oxygen from 20% to more physiological levels [6] (i.e. hypoxic culturing), influences MSC behavior [7]. In culture, hypoxia inhibits cell senescence, increases proliferation and enhances differentiation of MSCs derived from different sources [8, 9]. Hypoxia has been shown to impact the paracrine effects of MSCs via modulation of secreted factors [7, 10]. The benefits of hypoxic culture preconditioning were also demonstrated, in vivo, as hypoxic cultured MSCs better performed after transplantation in an ischemic environment, promoting wound and diabetic fracture healing [11–13]. Moreover, hypoxic cultured cells demonstrated enhanced survival, migration, angiogenesis and engraftment in vivo into a variety of tissues [7].

Oxygen partial pressure and hypoxic culture conditions have been shown to modulate signaling pathways. Hypoxia inducible factors HIF-1α and HIF-2α are transcription factors that are stabilized in the presence of low oxygen partial pressure and represent hypoxia key downstream effectors. They account for a significant fraction of the transcriptional control by hypoxia and hypoxia-inducible mRNAs (including those involved in angiogenesis, metabolism and apoptosis) [14, 15]. Few studies have performed genome-wide gene expression analysis comparing human bone marrow derived MSCs cultured under atmospheric oxygen partial pressure (20% O_2_) versus severe hypoxic (0.5% O_2_) to moderate hypoxic (5% O_2_) oxygen partial pressures [16–19]. Most studies have compared gene expression profiles obtained after short-term exposure to hypoxic culture conditions (24–72 h) and led to the identification of hundreds of dysregulated genes by hypoxia.

In the present study, we compared the properties of human bone marrow-derived mesenchymal stem cells after culture (i.e. 3 passages or 10–12 population doublings) in atmospheric (20% O_2_) versus hypoxic (5% O_2_) culture conditions from the time of isolation. The study was performed on cells that were isolated and cultured using materials and protocols in accordance with the manufacturing of MSCs for cellular therapies in human applications. We evaluated the potential benefit of moderate hypoxic culturing on hBMMSC properties and fitness and performed whole-genome expression analysis using mRNA sequencing technology. We found that only a small subset of genes expressed significant dysregulation in hypoxic conditions with only minor overlap with previously published studies and identified genes that were not previously associated with hypoxia. Moreover, our data highlights the benefits of hypoxic culturing on hBMMSC fitness and transcriptome and its impact on mesenchymal stem cell-based therapies with a particular emphasis for orthopedic applications such as chronic lower back pain.

## Methods

### Isolation of mesenchymal stem cells from human whole bone marrow

Three lots (three independent donors) of fresh human anticoagulated whole bone marrow were purchased from Lonza with each lot of bone marrow consisting of a volume of 15 ml. The bone marrow was diluted 1:1 by addition of 15 ml of calcium and magnesium free Dulbecco’s phosphate-buffered saline (DPBS, Gibco) and filtered through 100 µm cell strainer (Corning) to remove bone fragments. hBMMSC isolation was performed by density gradient. In a 50 ml conical, the 30 ml of diluted bone marrow were layered on top of 15 ml of Ficoll-Paque™ PREMIUM 1.073 (GE Healthcare). After centrifugation at 600*g* for 35 min at room temperature (18−22 °C) in a swinging bucket with the centrifuge brake off, the mononuclear cellular fraction was collected and washed twice with DPBS. Cells were finally pelleted at 500*g* for 5 min at room temperature, resuspended in 30 ml of growth medium (GM) and plated in a 225 cm^2^ flask.

### Cell culture and differentiation

Human bone marrow-derived mesenchymal stem cells were expanded in GM composed of Dulbecco’s modified Eagle’s medium (DMEM) low glucose (Gibco), supplemented with 10% human platelet lysate (Xcyte™ Plus Xeno-Free Supplement, iBiologics), 1% GlutaMAX™ Supplement (Gibco), 1% minimum essential medium non-essential amino acids (MEM-NEAA, Gibco), 100 units/ml of penicillin and 100 µg/ml of streptomycin (Gibco). Cells were cultured at 37 °C, 95% humidity and 5% CO_2_ in normoxia (20% O_2_) or hypoxia (5% O_2_). Cells were seeded at a density of 3500 cells/cm^2^ and medium was replaced every other day. Cells were subcultured before they reached confluence (80–90% confluence) using TrypLE (Gibco).

Adipocyte and osteoblast differentiation were induced 2 days after cells reached 100% confluency by replacing the GM with either the StemPro™ Adipogenesis Differentiation Kit (Gibco) or the StemPro™ Osteogenesis Differentiation Kit (Gibco). Differentiation was performed in normoxic conditions and medium was replaced every other day for 15 days.

Chondrocyte differentiation was performed in three-dimensions in atmospheric conditions. hBMMSC aggregates were formed in 15 ml polypropylene conicals by pelleting a suspension of 5 × 10^5^ cells in GM at 700*g* for 5 min. The GM was removed and the cellular aggregates were differentiated using the StemPro Chondrogenesis Differentiation Kit (Gibco). The differentiation medium was replaced twice a week for 21 days.

### Clonogenic assay

Proliferating hBMMSC were seeded at 100 cells per 100 mm dish (1.8 cells per cm^2^) in GM. GM was replaced every other day for 10 days, at which time colonies were formed. Colonies were fixed with 4% paraformaldehyde for 10 min, washed twice with deionized water and stained with a solution of 0.05% crystal violet in deionized water for 15 min at room temperature for visualization. Dishes were rinsed 3 times with tap water to remove the background stain and colonies were imaged and quantified.

### RNA isolation and quantitative polymerase chain reaction

Total RNA was isolated using Qiagen miRNeasy Mini Kit (Qiagen) according to manufacturer’s instruction and quantified using the NanoVue spectrophotometer (GE). cDNA was synthesized from 1 µg of total RNA in 20 µl reactions using the QuantiTect Reverse Transcription Kit (Qiagen) following manufacturer’s instruction. Quantitative PCR reactions were carried out in 20 µl using the TaqMan Fast Advanced Master Mix (Applied Biosystems), and TagMan gene expression assay probes (Applied Biosystems) on the QuantStudio 6 Flex Real-Time PCR system. Expression values were calculated as ∆∆CT using TBP as the reference. The TaqMan gene expression assays used the following: adipocyte markers comprising of FABP4, adipsin and CEBPa; osteoblast markers comprising of ALPL, CBFA1 and osteocalcin; chondrocyte markers comprising of Sox9, COL1A1, COL2A1 and ACAN.

### Whole-transcriptome RNA sequencing

RNA sequencing was carried out by SeqWright Genomic Services (Houston, Texas). Total RNA isolated, as described above, were quantified and assessed for quality by spectrophotometric measurement and agarose gel analysis. The mRNA library was prepared from 1 µg of total RNA using the illumina TruSeq RNA Sample Preparation Kit v2. After cluster generation, sequencing was performed on the Illumina HiSeq 2500 instrument in multiplex with 2 × 100 base pair read lengths for a total of 2 × 40 million reads per sample. Data was aligned to hg19 using Tophat-2.0.13 and analyzed using Cufflinks-2.1.1

### Flow cytometry analysis

Mouse monoclonal phycoerythrin (PE) conjugated anti-human CD90 (clone 5E10), mouse monoclonal APC conjugated anti-human CD105 (clone 43A3), mouse monoclonal pacific blue conjugated anti-human CD45 (clone HI30), Mouse monoclonal phycoerythrin (PE) conjugated anti-human CD73 (clone AD2), Mouse monoclonal phycoerythrin (PE) conjugated anti-human CD49b (clone p1E6-C5), and mouse monoclonal PerCP/Cy5.5 anti-human CD34 (clone 581) antibodies were purchased from BioLegend and used according to manufacturer’s instructions. Proliferating cells were collected from tissue culture flasks using TrypLE (Gibco) and resuspended in GM. Cells were washed with DPBS (Gibco) and 5 × 10^5^ cells were aliquoted for immunostaining. Immunostaining was performed using antibodies diluted in 100 µl of staining buffer composed of DPBS, 1% (V/V) bovine growth serum (BGS) and 0.1% (W/V) sodium azide for 30 min on ice. Cells were washed with ice cold DPBS and the analysis was performed on a Dual Fortessa 2 flow cytometer (BD Biosciences).

### Statistical analysis

Data from clonogenic assays, cellular identity by flow cytometry and gene expression by quantitative PCR were analyzed by two-tailed paired Student’s t-test. Data from mRNA sequencing were aligned to hg19 using Tophat-2.0.13 and analyzed using Cufflinks-2.1.1.

## Results

Determining adequate cellular culture conditions is critical in order to develop effective cellular therapies. In the present study, we compared the properties of human bone marrow-derived mesenchymal stem cells (hBMMSCs) cultured in atmospheric (20% O_2_) versus moderate hypoxic (5% O_2_) conditions from the time of isolation and evaluated the benefit of hypoxic culturing for orthopedic applications. Human mononuclear cell populations were freshly isolated from three independent anticoagulated whole bone marrow by density gradient. Immediately after isolation, each sample was split into two equal parts, plated in atmospheric or hypoxic conditions and cultured side by side for 3 passages (~ 10–12 population doublings). Throughout the study, all experiments were performed on cells cultured for 3 passages. Human bone marrow-derived mesenchymal stem cells (hBMMSCs), like other tissue specific MSCs, display stem cell characteristics such as multipotency, clonogenicity and specific surface marker expression commonly used to determine stem cell fitness and identity, as defined by the International Society for Cellular Therapy [20].

### Hypoxic culturing does not impact the identity of hBMMSCs

**Fig. 1:**
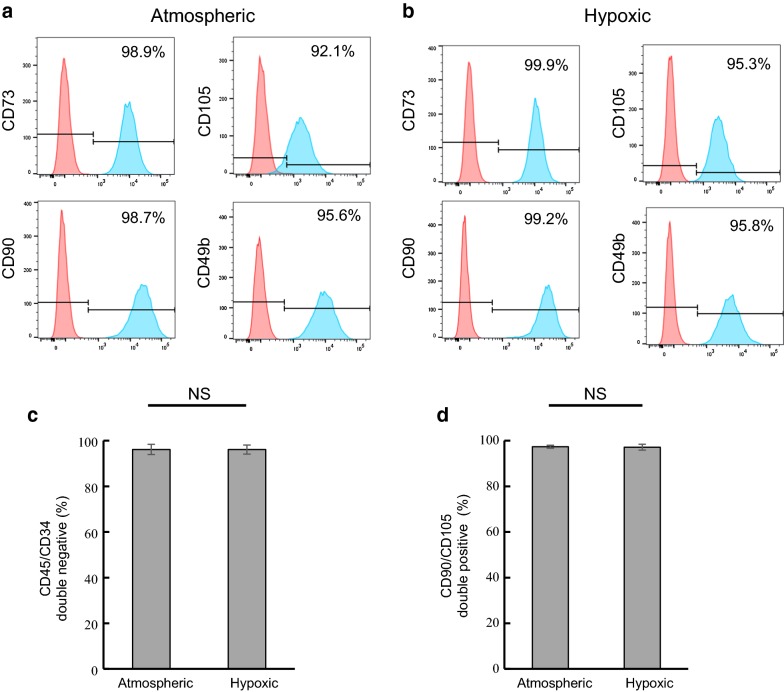
hBMMSCs cultured in atmospheric and hypoxic conditions display similar identity. Sample-matched hBMMSCs cultured in atmospheric or hypoxic conditions for 3 passages from the time of isolation were tested for the expression of surface markers characteristic of MSCs (CD90, CD105, CD73 and CD49b) and the absence of expression of hematopoietic markers (CD34 and CD45) by flow cytometry. **a**, **b** Representative dot plots of atmospheric cultured (**a**) and hypoxic cultured (**b**) hBMMSCs are presented. Positive for CD90, CD105, CD73 and CD49b whereas quadrant Q3-1 display cells that are double negative for CD34 and CD45. **c**, **d** Quantification of the percentage of cells that are CD34/CD45 double negative (**c**) and CD90/CD105 double positive (**d**) in hBMMSCs cultured in atmospheric and hypoxic conditions. Data represent mean ± SD, n = 3 independent sample-matched human BMMSCs. Two-tailed paired Student’s t-test: non-significant (NS) *p* value > 0.05

The expression of surface markers characteristic of MSCs, such as CD90, CD105, CD73 and CD49b and the lack of expression of hematopoietic markers, such as CD34 and CD45, was performed by flow cytometry analysis. As shown in Fig. 1, hBMMSCs are positive for CD90 and CD105 (≈ 97%) and negative for CD34 and CD45 (~ 96%) and display the surface markers characteristic of MSCs [20]. No significant difference in the expression of these markers was observed between atmospheric (20% O_2_) and hypoxic (5% O_2_) cultured hBMMSCs (Fig. 1c, d). Therefore, the oxygen partial pressure did not impact the identity of the cells based on the surface markers tested.

### Hypoxic culturing impact on cell growth and clonogenicity

**Fig. 2:**
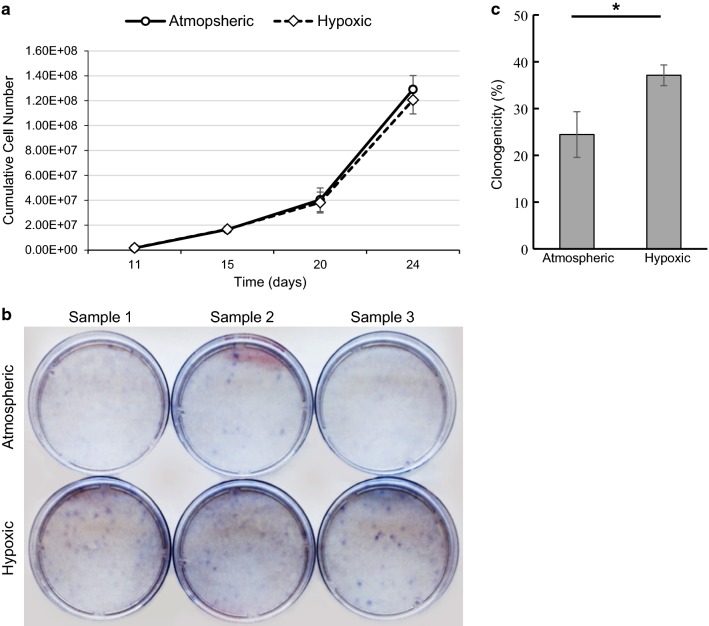
Hypoxic culturing increase the clonogenicity of hBMMSCs. Sample-matched hBMMSCs cultured in atmospheric or hypoxic conditions for 3 passages from the time of isolation were tested for proliferation (**a**) and clonogenicity (**b**, **c**) using the CFU-F assay (colony forming unit-fibroblasts). **a** Growth curves representing cumulative cell numbers over time are presented. Days in culture are relative to day 0 corresponding to hBMMSC isolation. Data represent mean ± SEM of n = 3 independent sample-matched hBMMSCs. **b**, **c** Cells were plated at very low density in their respective culture conditions and formed colonies were visualized by crystal violet staining. **b** Micrograph showing representative colony formation in 3 independent sample-matched atmospheric cultured and hypoxic cultured hBMMSCs. **c** Quantification of **b**. Data represent mean ± SD, n = 3 independent sample-matched hBMMSCs tested in triplicates. Two-tailed paired Student’s t-test *p value ≤ 0.05

Freshly isolated hBMMSCs were quiescent and became activated and started proliferating around day 3 or 4 in culture using human platelet lysate as a source of growth factors. On average, 11 days passed from the time of isolation to the first passage. Cells were passaged for the first time when cell clusters reached approximately 80–90% confluency. Once fully proliferating, population doubling times were approximately 24 h for both atmospheric and hypoxic cultured hBMMSCs. No difference in cell proliferation was observed between atmospheric and hypoxic cultured cells (Fig. 2a).

Clonogenicity, or the ability of single cells to grow into colonies, is another property of MSCs and is considered an indicator of stem cell fitness. We compared the clonogenicity of hBMMSCs cultured in atmospheric versus hypoxic conditions from the time of isolation using the CFU-F assay (colony forming unit-fibroblasts). As shown in Fig. 2b, c, the percentage of colony formation was significantly greater in hBMMSCs cultured in hypoxic conditions (37.1%) versus cells cultured in atmospheric conditions (24.4%) representing a 50% increase in clonogenicity in hypoxic cultured cells. These results are in accordance with data from other published studies indicating that hypoxic culturing in 5% oxygen partial pressure increased the clonogenicity of hBMMSCs, as compared to cultures in atmospheric oxygen partial pressure (20%) [21].

### Hypoxic culturing alter the differentiation potential of hBMMSCs

**Fig. 3:**
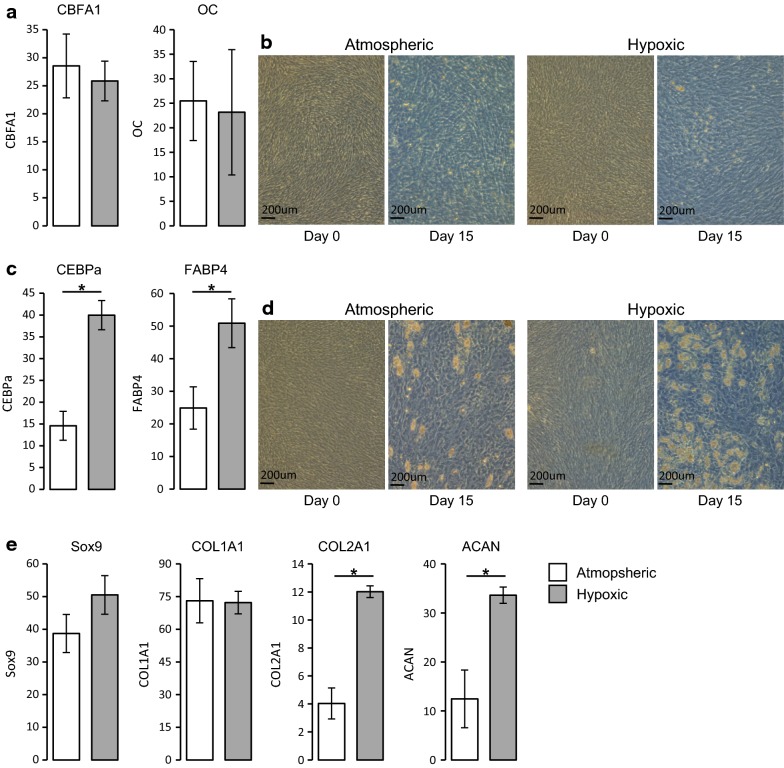
Hypoxic culturing increase the differentiation potential of hBMMSCs. Sample-matched hBMMSCs cultured in atmospheric or hypoxic conditions for 3 passages from the time of isolation were tested for their ability to differentiate into osteoblast (**a**, **b**), adipocyte (**c**, **d**) and chondrocyte (**e**). **a**, **c**, **e** hBMMSCs were able to undergo tri-lineage differentiation regardless of oxygen pressure, but demonstrated increased chondrocyte specific differentiation under hypoxia, specifically COL2A1 and ACAN. Relative gene expression by qPCR. **a** Expression of CBFA1 and osteocalcin (OC) 15 days after differentiation in osteogenic medium. **c** Expression of CEBPa and FABP4 15 days after differentiation in adipogenic medium. **e** Expression of Sox9, COL1A1, COL2A1 and ACAN 21 days after differentiation in chondrogenic medium. Data represent mean ± SEM, n = 3 independent sample-matched hBMMSCs. Two-tailed paired Student’s t-test *p value ≤ 0.05. **b**, **d** Representative micrographs of undifferentiated hBMMSCs (day 0) and hBMMSCs 15 days after differentiation into osteoblasts (**b**) or adipocytes (**d**)

Differentiation potential towards osteogenic, adipogenic and chondrogenic lineages were also evaluated. Differentiation of both atmospheric and hypoxic cultured cells were all performed in atmospheric culture conditions in order to eliminate the potential direct impact of the oxygen partial pressure on the differentiation process [22, 23]. hBMMSCs were able to differentiate into the tri-lineages, regardless of the oxygen partial pressure used during in vitro expansion. As shown in Fig. 3, no difference was observed between atmospheric-cultured and hypoxic cultured hBMMSCs in their ability to differentiate into osteoblasts. No difference was observed in the expression of the key transcription factor core-binding factor subunit alpha-1 (CBFA1) or the expression of the bone matrix associated gene osteocalcin (OC) (Fig. 3a, b). However, as visualized by the accumulation of lipids and the expression of the transcription factor CCAAT/enhancer binding protein alpha (CEBPa) and the fatty acid binding protein 4 (FABP4), hypoxic cultured hBMMSCs ability to differentiate into adipocytes was significantly higher than that of sample-matched atmospheric cultured cells (Fig. 3c, d). Interestingly, hypoxic culturing slightly increased the expression of the chondrocyte transcription factor SRY-box 9 (Sox9) and significantly increased the expression of the cartilage matrix associated genes collagen type II alpha 1 chain (COL2A1) and aggrecan (ACAN), but not collagen type I alpha 1 chain (COL1A1) (Fig. 3e). Therefore our results and as previously demonstrated by other studies, hypoxic culturing increases the chondrogenic potential of hBMMSCs [24, 25].

### Transcriptome analysis comparing cultures in atmospheric and hypoxic conditions

**Fig. 4:**
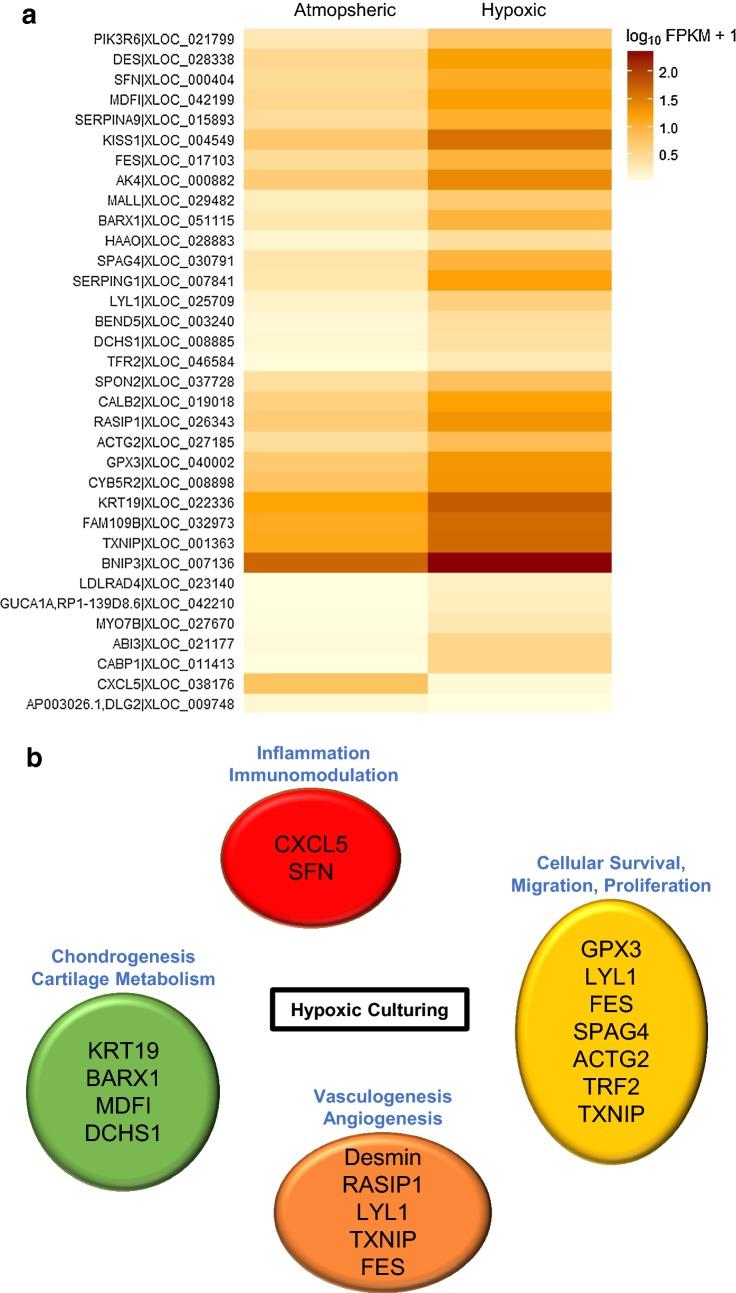
Whole transcriptome of atmospheric versus hypoxic cultured hBMMSCs. Transcriptomes of sample-matched hBMMSCs cultured in atmospheric or hypoxic conditions from the time of isolation and for 3 passages were analyzed using mRNA sequencing. **a** Heat map of all significant genes that are differentially expressed between atmospheric and hypoxic conditions is presented. Data represent the average of fragments per kilo base of exon per million fragments mapped (FPKM), n = 3 independent sample-matched hBMMSCs. **b** Schematic representing functional clustering of the differentially expressed genes

In order to identify key differences in gene expression between atmospheric and hypoxic cultured hBMMSCs, we performed a whole-transcriptome analysis by mRNA sequencing. RNA was isolated from undifferentiated, proliferating sample-matched atmospheric and hypoxic cultured hBMMSCs after 3 passages in culture corresponding to approximately 24 ± 5 days from the time of isolation or 10–12 population doublings. On average, hBMMSCs expressed a total of 19,729.8 genes. No significant difference in the total number of genes expressed was observed between atmospheric cultured and hypoxic cultured hBMMSCs with 20,181.0 ± 488.9 and 19,278.7 ± 1874.9 genes expressed, respectively. Surprisingly, out of the 19,729.8 genes expressed, only 34 genes were identified as significantly differentially expressed between the two culture conditions. Thirty-two (32) out of the 34 genes were upregulated in hypoxic culture conditions (Fig. 4 and Table 1). No difference in splice variants, isoforms, or gene fusion were observed between the two culture conditions. Among the differentially expressed genes, we identified genes previously shown to be regulated by hypoxia, such as BNIP3 [26], SPAG4 [27, 28], TRF2 [29] and TXNIP [30]. When genes were clustered based on biological functions, the differentially expressed genes identified were found to be involved in biological processes that are important to certain cell therapies such as: (1) chondrogenesis and cartilage metabolism; (2) inflammation and immunomodulation; (3) cellular survival, migration and proliferation; and (4) vasculogenesis and angiogenesis.

**Table 1:** Differentially expressed genes identified by mRNA sequencing

Gene symbol	Entrez gene name	Atmospheric (FPKM)	Hypoxic (FPKM)	Log_2_ (fold change)	p-value	q-value
Genes upregulated in hypoxia						
SFN	Stratifin	1.97	11.55	2.55	1.00E−04	4.47E−02
AK4	Adenylate kinase 4	3.64	23.81	2.71	5.00E−05	2.62E−02
TXNIP	Thioredoxin interacting protein	12.61	43.98	1.80	5.00E−05	2.62E−02
BEND5	BEN domain containing 5	0.28	1.70	2.59	5.00E−05	2.62E−02
KISS1	KiSS-1 metastasis-suppressor	4.29	38.05	3.15	5.00E−05	2.62E−02
BNIP3	BCL2 interacting protein 3	47.81	215.64	2.17	5.00E−05	2.62E−02
SERPING1	Serpin family G member 1	1.11	14.97	3.75	5.00E−05	2.62E−02
DCHS1	Dachsous cadherin-related 1	0.32	1.60	2.35	5.00E−05	2.62E−02
CYB5R2	Cytochrome b5 reductase 2	5.14	19.47	1.92	5.00E−05	2.62E−02
CABP1	Calcium binding protein 1	0.08	2.49	4.93	5.00E−05	2.62E−02
SERPINA9	serpin family A member 9	1.88	10.53	2.48	5.00E−05	2.62E−02
FES	FES proto-oncogene, tyrosine kinase	1.88	8.97	2.25	5.00E−05	2.62E−02
CALB2	Calbindin 2	2.97	14.79	2.32	5.00E−05	2.62E−02
ABI3	ABI family member 3	0.18	2.54	3.84	5.00E−05	2.62E−02
PIK3R6	Phosphoinositide-3-kinase regulatory subunit 6	1.04	4.70	2.17	5.00E−05	2.62E−02
KRT19	Keratin 19	14.18	57.12	2.01	5.00E−05	2.62E−02
LDLRAD4	Low density lipoprotein receptor class A domain containing 4	0.08	0.62	2.91	5.00E−05	2.62E−02
LYL1	LYL1, basic helix-loop-helix family member	0.45	3.08	2.78	5.00E−05	2.62E−02
RASIP1	Ras interacting protein 1	3.72	19.51	2.39	5.00E−05	2.62E−02
ACTG2	Actin, gamma 2, smooth muscle, enteric	1.79	6.37	1.83	1.00E−04	4.47E−02
MYO7B	Myosin VIIB	0.11	1.02	3.23	5.00E−05	2.62E−02
DES	Desmin	2.26	15.77	2.80	5.00E−05	2.62E−02
HAAO	3-Hydroxyanthranilate 3,4-dioxygenase	0.37	1.71	2.20	1.00E−04	4.47E−02
MALL	mal, T-cell differentiation protein like	0.72	4.07	2.51	5.00E−05	2.62E−02
SPAG4	Sperm associated antigen 4	1.22	8.56	2.81	5.00E−05	2.62E−02
FAM109B	Family with sequence similarity 109 member B	11.87	42.81	1.85	5.00E−05	2.62E−02
SPON2	Spondin 2	1.61	5.73	1.83	1.00E−04	4.47E−02
GPX3	Glutathione peroxidase 3	4.14	18.82	2.19	1.00E−04	4.47E−02
MDFI	MyoD family inhibitor	2.38	15.85	2.74	5.00E−05	2.62E−02
GUCA1A	Guanylate cyclase activator 1A	0.09	0.68	2.99	5.00E−05	2.62E−02
TFR2	Transferrin receptor 2	0.15	0.93	2.67	5.00E−05	2.62E−02
BARX1	BARX homeobox 1	1.10	8.74	3.00	5.00E−05	2.62E−02
Genes downregulated in hypoxia						
CXCL5	C-X-C motif chemokine ligand 5	5.22	0.21	− 4.66	5.00E−05	2.62E−02
DL G2	Discs large MAGUK scaffold protein 2	0.27	0.05	− 2.32	5.00E−05	2.62E−02

Fold changes represent hypoxia/normoxia FPKM values. p-value is the uncorrected p-value of the test statistic. q-value is the false discovery rate (FDR)-adjusted p-value of the test statistic. Statistical significance is based on the FDR-adjusted p-value

Hypoxic cultured hBMMSCs expressed higher levels of genes involved in chondrogenesis and cartilage metabolism. BARX1 (eightfold increased in hypoxia) is a transcription factor necessary for the proliferation of the ectomesenchyme and the osteochondroprogenitor condensation during development [31]. It has been linked with the chondrogenic potential of human embryonic stem cells [32, 33], clearly indicating a correlation between high level of BARX1 and chondrogenic potential. MyoD family inhibitor (MDFI) expression was increased by sevenfold in hypoxic conditions. MDFI is a transcription factor that functions as a negative regulator of the myogenic family of proteins and has been shown to play an important role in trophoblast and chondrogenic differentiation [34]. Dachsous cadherin-related 1 (DCHS1) expression is up regulated in hypoxic conditions by fivefold and is a cellular adhesion molecule member of the cadherin superfamily. Lack of DCHS1 results in developmental defects of the early sclerotome associated with fewer chondrogenic cells within the developing vertebral body [35]. Keratin 19 (KRT19) expression showed a fourfold increase in hypoxic cultured samples and is an intermediate filament protein that is part of the keratin family. KRT19 has been identified as a nucleus pulposus (NP) specific marker of the human intervertebral disc (IVD) when compared to annulus fibrosus cells and articular cartilage cells [36, 37]. KRT19 is considered a marker of discogenesis, in vitro [38], and has been shown to be co-expressed with chondrogenic markers, such as Aggrecan, Sox-9 and Col2A1, during isolation and immortalization of NP cells from human non-degenerated IVD [39]. Interestingly, KRT19 expression has been associated with notochord-like cells in non-degenerated IVD [37] and its protein expression decrease with NP degeneration in humans. KRT19 expression is also altered in both rodent and human aged IVD [36, 40]. Even though the transcription factor Sox9, a key regulator of chondrocyte differentiation, was expressed at similar levels between atmospheric and hypoxic cultured cells, the differentially expressed genes discussed above tend to indicate that hypoxic cultured hBMMSCs are more prone to give rise to chondrogenic cells than their atmospheric cultured counterparts.

We also observed differential expression of genes involved in inflammation and immunomodulation. The pro-inflammatory C-X-C motif chemokine 5 (CXCL5) expression levels were 25 times lower in hypoxic cultured cells and close to the detection limit (FPKM ≤ 1), and therefore, hBMMSCs are more immune privileged than atmospheric cultured cells. Stratifin (SFN) expression, however, showed a sixfold increase in hypoxic conditions. SFN is an adaptor protein that modulates the activity of the binding partner and has been shown, in stratified epithelial cells, to be a potent anti-fibrogenic and anti-inflammatory factor that regulates extracellular matrix deposition [41].

Hypoxic cultured cells displayed higher levels of pro-survival, pro-proliferation and pro-migration genes than hBMMSCs cultured in atmospheric conditions. Cells cultured in hypoxic conditions showed a 4.5-fold increase in the expression of the glutathione peroxidase 3 (GPX3), an enzyme that protects cells and proteins from oxidative damage by catalyzing the reduction of hydrogen peroxide, lipid peroxides, and organic hydroperoxide by glutathione. This detoxification enzyme has also been shown to be a crucial factor for human skeletal muscle precursor cell survival [42]. Similarly, the expression of the gene coding for the thioredoxin interacting protein (TXNIP), a thiol-oxidoreductase enzyme, displayed a 3.5-fold increase in hypoxic conditions. TXNIP is a major regulator of intracellular reactive oxygen species (ROS) and protects cells from oxidative stress. Other genes identified herein have been linked to cellular survival and proliferation in different cell types [29, 43–47]. These genes include the basic helix-loop-helix transcription factor lymphoblastic leukemia associated hematopoiesis regulator 1 (LYL1, sevenfold increase in hypoxia), the tyrosine-specific protein kinase feline sarcoma proto-oncogene (FES, fivefold increase in hypoxia), and the Transferrin receptor 2 (TFR2, sixfold increase in hypoxia). Hypoxic cultured cells also expressed higher levels of the actin gamma 2 (ACTG2) and the spermatid-specific gene 4 (SPAG4) genes, both of which were shown to increase cellular migration and the latter was shown to protect chromosomal integrity during cellular division in hypoxic conditions [27, 48–50].

Among the differentially expressed genes, a subset of genes are involved in vasculogenesis and angiogenesis. Desmin (increased by sevenfold in hypoxia) is an endothelial cell marker and is expressed in pericytes [51], elongated mural support cells that extend along endothelial cells regulate vessel permeability, vessel diameter and endothelial cell proliferation through both paracrine signaling and direct contact with endothelial cells [52, 53]. FES and Ras interacting protein 1 (RASIP1) were both increased in hypoxic conditions by approximately fivefold. These genes contribute to endothelial cell function during angiogenesis, as well as endothelial cell morphogenesis and blood vessel tubulogenesis [45, 54–58]. LYL1, which controls the expression of the vascular growth factor angiopoietin-2 in endothelial cells [59] and TXNIP (required for VEGF-mediated VEGFR2 activation and angiogenic response [60]), were increased by sevenfold and 3.5-fold in hypoxic conditions, respectively.

## Discussion

Mesenchymal stem cells (MSCs) hold great potential to treat various degenerative diseases as evidenced by the fast growing number of preclinical and clinical stage studies published. MSCs are found at low frequency in adult tissues and in vitro amplification is required for the manufacture of MSC based cellular therapies in order to produce clinically therapeutic numbers [3, 4]. Controlling and optimizing culture conditions during MSC in vitro expansion is essential in order to manufacture potent cell therapeutics as these culture conditions impact the characteristics of the cells and their behavior after transplantation. In the present study, we evaluated the impact of hypoxic culturing on human bone marrow derived mesenchymal stem cells (hBMMSCs) isolated and cultured using materials and protocols in accordance with the manufacturing of MSCs for cellular therapies in human applications. We compared their cellular properties, as well as their transcriptome after culture (3 passages or 10–12 population doublings), in atmospheric (20% O_2_) or moderate hypoxic (5% O_2_) conditions from the time of isolation.

Maintaining hBMMSCs in a high degree of “stemness”, represented by self-renewal, differentiation potential and clonogenicity, is a prerequisite to the successful use of stem cells in regenerative medicine. Herein, we demonstrated that hypoxic culturing increased cellular clonogenicity confirming data from previous reports [18, 21, 61]. Moreover, we showed that culture in hypoxic conditions improved the differentiation potential of hBMMSCs towards adipocyte and chondrocyte lineages, whereas no effect were observed on osteoblast differentiation. Conflicting data has been published regarding the impact of hypoxia on MSC differentiation [7, 21, 22, 61, 62]. These discrepancies are likely multifactorial and include the heterogeneity of MSCs isolated from the bone marrow, the oxygen partial pressure used, as well as variations in growth and differentiation mediums. The oxygen partial pressures used during cellular expansion (cell growth) and differentiation appears to be a determinant factor [21, 22, 63]. Hypoxia has been shown to affect differentiation processes directly [63]. Moreover, Boyette et al. [21] described an inhibitory effect of hypoxia on osteogenesis, but only when cells were cultured and preconditioned in atmospheric culture conditions. In our study, cells isolated from each bone marrow sample were split and cultured in either atmospheric (20% O_2_) or hypoxic (5% O_2_) conditions, but differentiation processes were performed in atmospheric conditions in order to avoid the effect of hypoxia on cell fitness versus differentiation. It is interesting to note that even though atmospheric and hypoxic cultured cells have a similar cell surface phenotype, hypoxic cultured cells displayed higher clonogenicity and differentiation potential than cells cultured in atmospheric conditions. Other studies reported similar observations and, although it can’t be ruled out that a discriminating surface marker hasn’t been yet identified, it seems likely that oxygen partial pressure intrinsically modifies cell properties since different sets of markers were used in these studies covering higher numbers of surface markers [18, 19, 63].

Using mRNA sequencing to compare the transcriptome of cultured hBMMSC in atmospheric (20% O_2_) versus hypoxic (5% O_2_) conditions, we have identified 34 differentially expressed genes. These differentially expressed genes provide the first step towards understanding the potential benefit and mechanism of action of hypoxic cultured cells. Few previous studies compared whole genome expression profiles of hBMMSCs cultured in atmospheric versus hypoxic conditions and identified genes that were dysregulated by hypoxia [17–19]. These reports identified much larger numbers of genes dysregulated by hypoxia, ranging from 282 to 519 genes, than the ones identified in the present study (34 genes). Moreover, it is interesting to note that in these studies 45–51% of the dysregulated genes were upregulated in hypoxic conditions, which is in contrast to the 94% observed herein (32 upregulated genes out of the 34 dysregulated genes identified). These differences could be attributed to the heterogeneity in the hBMMSCs isolated even though the isolation processes of bone marrow-derived MSCs are well described and fairly standard. This could also be explained by the difference in oxygen partial pressure used, ranging from severe (0.5% O_2_) to moderate (5% O2) hypoxia, and by the duration of hypoxic culturing from short-term (24 h) to culture (3 passages or 24 ± 5 days) [17–19]. However, Basciano et al. [18] identified 519 dysregulated genes, despite using the same oxygen partial pressure (5% O_2_) as the one used in the present study, and performing hypoxic culture for up to 2 passages (or 21 days). One key difference between previous reports comparing whole genome expression of hBMMSCs and the present study is the method used to assess the transcriptome. Herein, we have used mRNA sequencing technology, whereas data from previous reports came from microarrays technologies. Although both methods have drawbacks and advantages [64], RNA-sequencing provides a far more precise measurement of levels of transcripts and their isoforms than other methods [65], which could therefore account for the discrepancies.

Chronic and inflammatory diseases of joints and the spine, including osteoarthritis and low back pain are major causes of disability. Low back pain is also the most common cause of disability among Americans between 45 and 65 years of age [66]. These diseases are multifactorial and associated with the degeneration of articular cartilage and intervertebral disc (IVD), in the case of osteoarthritis and low back pain, respectively. The IVD and the articular cartilage are two different types of cartilages but they share similar features in that they are avascular, aneural and alymphatic, limiting their self-repair potential. During degeneration, these tissues are characterized by a decrease in the number of specialized chondrocytes impairing the production of cartilage extracellular matrix proteins and leading to loss and remodeling of the extracellular matrix. These biochemical changes impact the biomechanical function of these tissues and their ability to respond to mechanical load, therefore allowing for more cartilage lesions to occur. Over time, local inflammation increases, vasculature at the periphery of these tissues decreases (diminishing the diffusion of nutrients) and nerve in growth is observed. This degenerative cycle inhibits regeneration and ultimately leads to pain [67, 68]. Previous studies have suggested that adipose-derived stem cells hold advantages over bone marrow-derived stem cells because adipose tissue is very abundant and readily accessible. From a research use perspective this maybe true, but for clinical application, bone marrow-derived stem cells are more easily translated into the clinic because there is no use of enzymatic separation, which the FDA imposes regulatory oversight. Human bone marrow-derived mesenchymal stem cells (hBMMSCs) hold great regenerative promises to replace or restore function of damaged tissues and organs. They have been studied extensively and are well characterized, being amongst the first mesenchymal stem cells to be discovered and translated clinically. Because of their inherent properties, such as anti-inflammatory, immune modulation and their high chondrogenic differentiation potential, they represent a highly regarded cell source for orthopedic applications such as articular cartilage or intervertebral disc (IVD) repair [67, 68]. In the present study, among the genes dysregulated by hypoxia, we identified genes involved in chondrogenesis, inflammation and immunomodulation, cellular survival, migration and proliferation, as well as genes involved in vasculogenesis and angiogenesis. Therefore, our data suggest that hypoxic culturing improves hBMMSCs inherent properties and might potentiate their action in vivo. Moreover, the oxygen partial pressures found in the bone marrow (hBMMSCs niche) range between 2 and 7% and the oxygen partial pressures described in the articular cartilage and the IVD are ranging from 1 to 5% [69, 70]. Therefore, expanding hBMMSCs under hypoxia in vitro rather than in atmospheric oxygen partial pressure conditions seems very intuitive and it has been proven beneficial [7, 71]. Some of the genes identified herein have previously been described to be regulated by hypoxia, such as BNIP3 [26], SPAG4 [27, 28], TRF2 [29] and TXNIP [30]. Stratifin (SFN) was identified for the first time as being regulated by hypoxia in hBMMSCs with a sixfold upregulation in hypoxic conditions versus atmospheric culture conditions. SFN is of particular interest for orthopedic applications since it is a potent anti-fibrotic and anti-inflammatory factor [41]. Increasing cellular expression of SFN by hypoxic culturing and overexpression or stimulation with soluble molecules could represent a new strategy to improve cellular therapeutics targeting orthopedic diseases.

Overall, based on our results and data from literature, expanding cells in hypoxic culture conditions impacts cell fitness and gene expression that may contribute to cell potency. Hypoxia may be of particular importance to select and condition cells that will be best suited for the targeted microenvironment in order to obtain an optimal therapeutic response.

## Conclusions

In conclusion, our study demonstrated that hypoxic culturing improves mesenchymal stem cell properties and positively influences whole genome expression profiles with respect to the development of cellular therapies in orthopedic applications using human bone marrow derived mesenchymal stem cells. Currently, BioRestorative Therapies has been authorized to commence (by the FDA) a Phase II clinical trial using hypoxic cultured bone marrow-derived mesenchymal stem cells to treat chronic lower back pain. With the results obtained in this study, we expect to see better patient outcomes using hypoxic cultured cells vs normoxic ones.

## Abbreviations

CAR: chimeric antigen receptor
MSC: mesenchymal stem cell
HIF: hypoxia inducible factor
hBMMSC: human bone marrow derived mesenchymal stem cell
DPBS: Dulbecco’s phosphate-buffered saline
GM: growth medium
MEM-NEAA: minimum essential medium non-essential amino acids
TBP: tata-box binding protein
FABP4: fatty acid binding protein 4
CEBPa: CCAAT/enhancer binding protein alpha
ALPL: alkaline phosphatase, liver/bone/kidney
CBFA1: core-binding factor subunit alpha-1
SOX9: sry-box 9
COL1A1: collagen type I alpha 1 chain
COL2A1: collagen type II alpha 1 chain
ACAN: aggrecan
BGS: bovine growth serum
CFU-F: colony forming unit-fibroblasts
OC: osteocalcin
SFN: stratifin
AK4: adenylate kinase 4
TXNIP: thioredoxin interacting protein
BEND5: BEN domain containing 5
KISS1: kiss-1 metastasis-suppressor
BNIP3: BCL2 interacting protein 3
SERPING1: serpin family G member 1
DCHS1: dachsous cadherin-related 1
CYB5R2: cytochrome b5 reductase 2
CABP1: calcium binding protein 1
SERPINA9: serpin family A member 9
FES: FES proto-oncogene, tyrosine kinase
CALB2: calbindin 2
ABI3: ABI family member 3
PIK3R6: phosphoinositide-3-kinase regulatory subunit 6
KRT19: keratin 19
LDLRAD4: low density lipoprotein receptor class A domain containing 4
LYL1: LYL1, basic helix-loop-helix family member
RASIP1: ras interacting protein 1
ACTG2: actin, gamma 2, smooth muscle, enteric
MYO7B: myosin VIIB
DES: desmin
HAAO: 3-hydroxyanthranilate 3,4-dioxygenase
MALL: mal, T-cell differentiation protein like
SPAG4: sperm associated antigen 4
FAM109B: family with sequence similarity 109 member B
SPON2: spondin 2
GPX3: glutathione peroxidase 3
MDFI: MyoD family inhibitor
GUCA1A: guanylate cyclase activator 1A
TFR2: transferrin receptor 2
BARX1: BARX homeobox 1
CXCL5: C-X-C motif chemokine ligand 5
DLG2: discs large MAGUK scaffold protein 2
FPKM: fragments per kilobase of exon per million fragments mapped
NP: nucleus pulposus
ROS: reactive oxygen species
